# From Pharmacy to Medicine

**Published:** 1915-05-15

**Authors:** 


### FROM PHARMACY TO MEDICINE.

There are such obvious relations between pharmacy and medicine that almost inevitably a certain number of men having obtained the one are seized with an ambition to reach the other. Hence, probably every year a certain small number of pharmacists become qualified medical practitioners, and as this achievement in itself argues some aspiration and industry above the average it is not surprising to find that such men are for the most part very successful in their new calling. Others, no doubt, there are who gaze at the promised land, but shrink from the wilderness. For some time there has been a somewhat ill-defined contention that the qualified pharmacist determined on the medical curriculum ought to be excused from certain parts of the examinations. He has already passed a preliminary examination in general knowledge and technical tests in dispensing, practical pharmacy, chemistry, and botany. Why, it is asked, should he be required to repeat these? This question has now been raised to some importance, as, we understand, it has been presented, no doubt in due and courteous form, by the Pharmaceutical Society to the General Medical Council. There is, we think, something to be said for the aspiring pharmacists, though even were the whole of their prayer granted no very substantial diminution of the obstacles in front of them would be effected. Perhaps relief from the preliminary examination would be the most substantial help, though if their own test of this order has the necessary qualities it can already gain recognition at the hands of the Medical Council and licensing bodies. To a qualified pharmacist the medical examinations in such subjects as practical pharmacy and chemistry and dispensing cannot mean a serious trial, for he must have had a more thorough training in these than has the average medical student. Even if he obtained relief from them it would not diminish the length of his period of study, and compared with what remains his difficulties would be but little diminished. Further, it has to be remembered that the General Medical Council cannot compel the licensing bodies to accept other examinations in place of their own tests, and, in any case, it is in the highest degree unlikely that the Universities, at least those who prescribe residence, would even listen to such a proposal. Their view, rightly or wrongly, is that the necessary avenue to an academic degree ought to include not merely examination tests, but the discipline and training of courses of study ordered in harmony with conditions which they themselves have formulated. The General Medical Council has no ability to modify these demands even supposing, which is in the highest degree unlikely, that it had the desire, and the Pharmaceutical Society cannot hope to succeed in such an aim. Hence it is impossible for the pharmacist who would be a physician to get much relief from the existing demands of the medical curriculum. His thorough training in some few of the numerous subjects which the curriculum includes will be of substantial help to him both in examination tests and in the more enduring tests of practical life; and he has presumably acquired in some degree a scientific outlook. If he cultivates the ambition and is of stout heart and good courage let him go forward by all means, and we doubt not he will succeed. But, like the rest of us, he must satisfy the necessary conditions, and obstacles exist to be overcome rather than to be conjured out of the way. Too anxious a desire in this latter direction recalls the fine gentleman who, but for the "vile guns, would himself have been a soldier."

